# Nutrition interventions for spine-related pain: A scoping review

**DOI:** 10.17305/bb.2024.11393

**Published:** 2024-11-08

**Authors:** Chelsey Hoffmann, Chloe Kom, Jordan Mackner, Leslie Hassett, Benjamin Holmes

**Affiliations:** 1 Department of Anesthesiology, Division of Pain Medicine, Mayo Clinic, Rochester, Minnesota, USA; 2 Mayo Clinic School of Health Science Dietetic Internship, Mayo Clinic, Rochester, Minnesota, USA; 3 University of Arizona College of Medicine-Phoenix, Phoenix, Arizona, USA; 4 Mayo Clinic Libraries, Mayo Clinic, Rochester, Minnesota, USA; 5 Department of Physical Medicine and Rehabilitation, Mayo Clinic, Rochester, Minnesota, USA

**Keywords:** Back pain, neck pain, spine pain, nutrition, supplement, diet

## Abstract

Multiple studies have been published regarding various nutritional supplements or interventions to improve chronic pain. However, many of these studies emphasized widespread pain and were not specific to the spine. Therefore, the primary objective of this scoping review was to evaluate available evidence related to nutritional supplementation or dietary strategies for spine-related pain. A comprehensive literature search was performed on October 11, 2022, and updated on May 2, 2024. Databases included: MEDLINE (PubMed), Embase, Cochrane Library, Scopus, and Web of Science. Results were limited to those published within the past 10 years, to English-language articles, and excluded animal studies. Of the 2,081 screened articles, 29 were included in the final review. Of these, 26 focused on the low back, one on the neck, and two referred to generalized “back” pain. The largest number of studies were found on vitamins D and B, specifically for low back pain. However, there were conflicting findings for both vitamins; therefore, further research is necessary before these can be confidently recommended to patients suffering from low back pain. Furthermore, this scoping review identified a lack of consistency in study design, population or sample size, and outcome measures among currently published studies with a primary focus on nutritional supplementation or dietary strategies for spine-related pain.

## Introduction

According to the World Health Organization, low back pain (LBP) affected 619 million people globally in the year 2020. Neck and mid-back pain are also common [1]. The high prevalence of spine-related pain from spondylosis, stenosis, vertebral compression fractures, or other etiologies has driven extensive research into treatment options. Additionally, there has been a recent increase in published literature investigating the relationship between nutrition and chronic pain. However, few of these studies focus specifically on spine-related pain, and to the authors’ knowledge, nutritional interventions for spine-related pain have not been comprehensively reviewed. Therefore, this scoping review was conducted to systematically map the research in this area and to highlight any existing knowledge gaps.

Patients with spine-related pain may seek advice or guidance from their healthcare providers regarding nutritional interventions or dietary supplements for pain relief. Providers should be aware of the most current evidence. The primary objective of this scoping review was to evaluate the available evidence related to nutritional supplementation or dietary strategies for spine-related pain. The secondary objective was to assess the impact of nutritional supplementation or dietary strategies on patient functionality, disability, and quality of life.

## Materials and methods

This study was designed as a scoping review to assess and understand the existing knowledge on nutritional supplementation or dietary strategies for spine-related pain. The term “spine-related pain” was used to encompass any painful etiology affecting the cervical, thoracic, or lumbar spine. A comprehensive search of several databases was performed on October 11, 2022, and updated on May 2, 2024. Results were limited to the English language and filtered for publication from the past ten years. The databases searched (and their content coverage dates) included Ovid MEDLINE(R) 1946 to Present and Epub Ahead of Print, In-Process & Other Non-Indexed Citations and Daily, Ovid Embase (1974+), Ovid Cochrane Central Register of Controlled Trials (1991+), Ovid Cochrane Database of Systematic Reviews (2005+), Web of Science Core Collection via Clarivate Analytics (1975+), and Scopus via Elsevier (1970+).

The search strategies were designed and conducted by a medical librarian, L. C. H., with input from the study investigators. Controlled vocabulary, supplemented with keywords, was used. Keywords included: lumbar pain, spine pain, back pain, nutrition therapy, dietary supplements, vitamins, and vitamin supplementation. The full search strategies can be found in Table S1.

**Figure 1.**
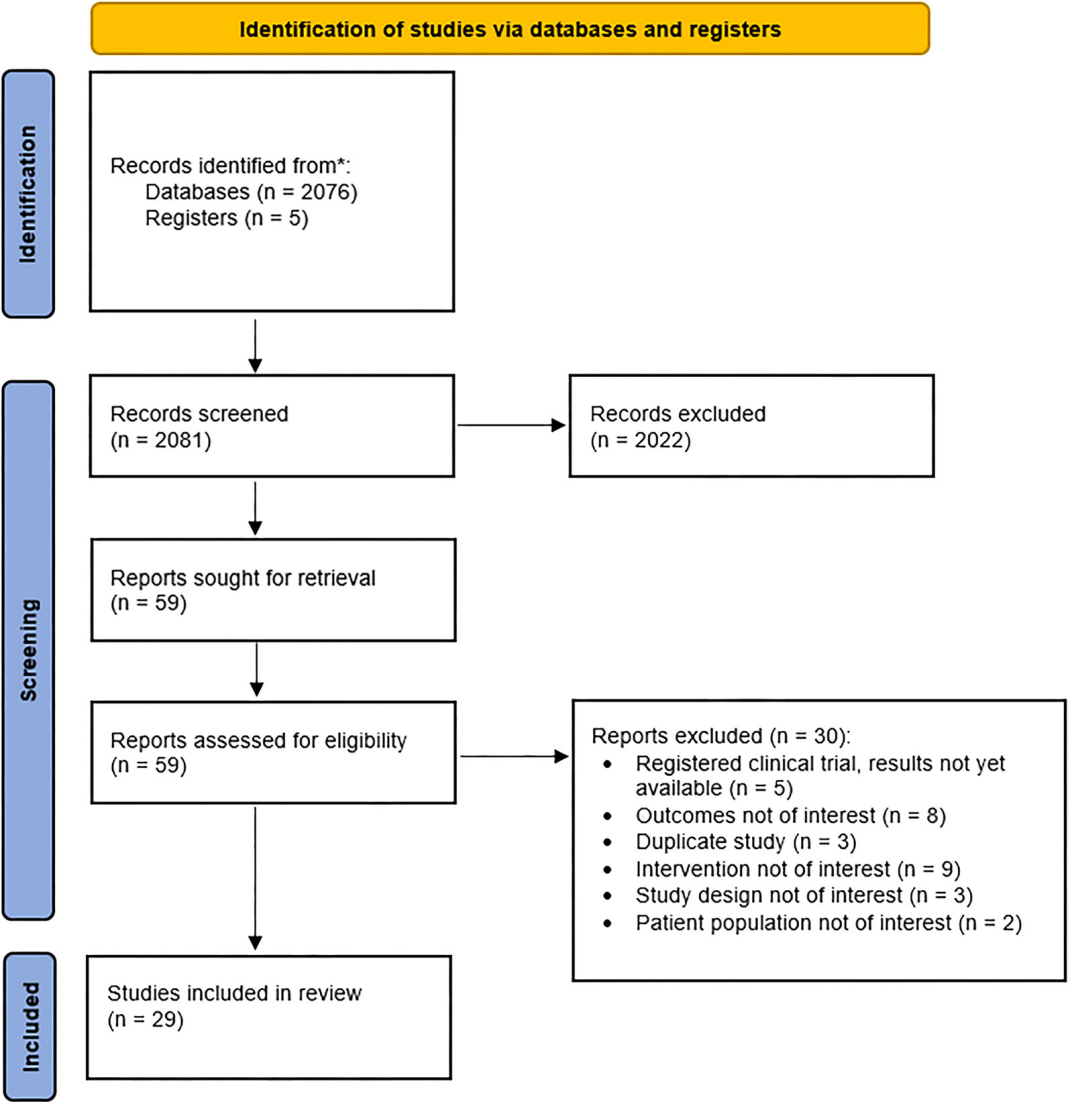
Search strategy and study selection.

Studies were included if the participants were adults aged 18 years or older and were suffering from spine pain (cervical, thoracic, or lumbar). The included study designs comprised randomized controlled trials (RCTs) with statistical analysis, non-randomized observational studies such as cohort studies, case-controlled studies, cross-sectional studies, case series, previous systematic reviews, and clinical trials. The primary outcome of interest was pain relief, measured using the numerical pain rating scale (NPRS), numerical pain scale (NPS), visual analog scale (VAS), or other validated screening instruments. Secondary outcomes of interest included patient functionality, disability, or quality of life, measured by the functional pain scale (FPS); Oswestry disability index (ODI); pain intensity, enjoyment of life, general activity (PEG-3) scale; patient-reported outcomes measurement information system (PROMIS); Bournemouth questionnaire; Roland-Morris disability questionnaire (RMDQ); neck disability index (NDI); or other validated functional, disability, or quality-of-life screening tools. Studies were excluded if the study design was unstated or unclear. Registered clinical trials without available results were also excluded. Additional exclusion criteria included animal studies, studies involving herbs, and studies that did not utilize validated pain or functionality screening instruments. Figure 1 depicts the complete study selection process.

Final search results were imported into Covidence. Two reviewers, Chloe Kom and Jordan Mackner, screened all studies. Where disagreements occurred, a third and final review was conducted by Chelsey Hoffmann. Benjamin Holmes provided consultation and expertise throughout the process. Given the scoping review design, the references of the included studies were not screened. A data chart was developed by two study authors, who independently reviewed the included studies and completed the chart. As the data chart (Excel spreadsheet) was completed, findings were discussed and iteratively updated throughout the study. Extracted data included the manuscript title, spinal region studied (i.e., cervical, thoracic, or lumbar), study design, citation, study population, intervention, control, outcome measures, and findings. During this process, it became evident that synthesizing the results would be most effective by grouping studies according to the anatomical spine region of focus (i.e., cervical spine, thoracic spine, lumbar spine, or generalized back pain) and the vitamin or supplement studied. Furthermore, extensive attention was given to whether the study outcome measures used validated pain or functional screening instruments (i.e., VAS, modified Oswestry disability questionnaire [MODQ], etc.).

## Results

A total of 2081 records were screened. After title and abstract screening, 2022 records were excluded based on the pre-specified inclusion and exclusion criteria. Of the 59 articles sought for retrieval and assessed for eligibility, 30 were excluded, leaving 29 articles for full-text review. Of the 29 reviewed articles, 26 focused on LBP, one on neck pain, and two on generalized “back” pain. Refer to Table 1 for a visual summary of the included studies. A more detailed summary of the findings is available in Table S2.

**Table 1:** Results summary

**Spine pain location**	**Intervention**	**# Studies found**
Low back	Vitamin D	8
Low back	Vitamin B	3
Low back	Alpha lipoic acid	2
Low back	Other*	13
Neck	Black soybean	1
Generalized back	Ossein-hydroxyapatite complex	1
Generalized back	Vitamin D	1
**Total studies: **		**29**

*Other interventions included: Telephone lifestyle advising and weight loss coaching, 24-h food recall, Noxiall (nutraceutical), bacillus subtilis var. natto products, magnesium, seal oil, collagen peptides, calcium carbonate, low-calorie diet, milk fat globule membrane and glucosamine, French maritime pine bark extract, lactobacillus rhamnosis, and intermittent diet.

### LBP

#### Vitamin D supplementation

Eight articles specifically investigated vitamin D supplementation, with or without additional supplements. Study designs included a prospective clinical trial (*n* ═ 1), RCT (*n* ═ 2), single-arm open-label clinical trial (*n* ═ 2), randomized, prospective, open-label cohort studies (*n* ═ 2), and a systematic review and meta-analysis (*n* ═ 1).

The first single-arm open-label study involved 68 participants who received 60,000 IU of oral vitamin D3 weekly for eight weeks [2]. At the conclusion of the study, 45 patients (66%) attained normal vitamin D levels post-supplementation. The mean (SD) VAS scores were 61 (19), 45 (19), and 36 (18) at two-, three-, and six-months post-supplementation, respectively, compared to a baseline VAS score of 81 (*P* < 0.001 at all time intervals). Using the MODQ, investigators also observed significant improvements in patients’ functional ability at two-, three-, and six-months post-supplementation compared to baseline [2]. Specifically, the mean (SD) MODQ at score baseline was 45, with a reduction to 36 (12) at two months, 31 (13) at three months, and 26 (10) at six months post-supplementation (*P* < 0.001 at all time intervals). Limitations included a small sample size and the lack of a control group for comparison.

The second single-arm open-label study investigated the effects of vitamins D, C, and E, and zinc supplementation in patients suffering from chronic LBP [3]. A total of 20 patients participated and were supplemented with an active vitamin D3 sachet of 60,000 IU orally every week for eight weeks. If a patient’s baseline serum vitamin D level was <5 ng/mL, they were given 60,000 IU daily orally for the first five days, followed by 60,000 IU weekly for the next eight weeks. Additional supplementation included vitamin C 1000 mg, Vitamin E 100 IU, and zinc 25 mg. Each of the additional supplements was taken daily for eight weeks. Outcome measures included the McGill pain questionnaire (MPQ), the finger floor test (FFT), the RMDQ, and the fear-avoidance beliefs questionnaire (FABQ). The investigators concluded that vitamins D, C, and E, and zinc may be effective in the treatment of chronic pain, based on the comparison of pre-supplementation and post-supplementation outcomes. [MPQ: pre-supplementation ═ 50.55 ± 6.03, post-supplementation ═ 23.45 ± 5.35; FFT: pre-supplementation ═ 5.5 ± 2.35 cm, post-supplementation ═ 2.45 ± 0.89 cm; RMDQ pre-supplementation ═ 14.9 ± 2.38, post-supplementation ═ 6.95 ± 1.70; and FABQ: pre-supplementation ═ 2.45 ± 0.89, post-supplementation ═ 22.5 ± 5.73] [3]. Statistical significance was defined as “*P* < 0.05” [3]. Limitations include the small sample size (*n* ═ 20) and the simultaneous use of multiple supplements by study patients.

A randomized, prospective, open-label study of 84 patients with mechanical LBP investigated the effects of supplemental vitamin D at a dose of 60,000 IUs/day for ten consecutive days, administered in the form of granules, nano syrup, or soft gel capsules. Pain and functional disability were assessed using the VAS and the MODQ. Specifically, VAS was analyzed by the chi-square test. The researchers found a significant difference between pre- and post-supplementation VAS scores across all three treatment groups; notably, the nano syrup formulation resulted in significantly better results [4] [granule pre-VAS vs post-VAS chi-square ═ 137.64, contingency co-efficient ═ 0.909, DF ═ 105, *P* value ═ 0.02; nano syrup pre-VAS vs post-VAS chi-square ═ 86.15, contingency co-efficient ═ 0.87, DF ═ 25, *P* value < 0.0001; and soft gel capsule pre-VAS vs post-VAS chi-square ═ 122.51, contingency co-efficient ═ 0.905, DF ═ 48, *P* value < 0.0001]. The researchers also used a paired *t*-test to analyze patient MODQ scores before and after treatment, finding the differences to be statistically significant [granule subgroup *t* ═ –10.93, DF ═ 28, *P* < 0.0001; nano syrup subgroup *t* ═ –11.81, DF ═ 27, *P* < 0.0001; and soft gel capsule subgroup *t* ═ –9.4, DF ═ 26, *P* < 0.0001]. A similar study in patients with chronic LBP and vitamin D deficiency was performed using the same three variations of vitamin D (granules, nano syrup, or soft gel capsules) for each of the three study groups [5]. However, the supplementation period was extended to 12 weeks. The results showed that vitamin D levels increased significantly across all study groups, with the nano syrup formulation group achieving the greatest increase. The nano syrup group also experienced a more significant reduction in LBP (paired *t*-test VAS ═ –7.2; *P* value < 0.001) [5].

An RCT pilot study of 51 patients investigated the effects of vitamin D supplementation in patients with chronic LBP secondary to lumbar spinal stenosis [6]. Patients received 200,000 IU cholecalciferol intramuscularly, with an additional 800 IU oral vitamin D after 12 weeks if their serum 25OH-Vit D levels remained between 20 and 30 ng/mL [6]. The vitamin D supplementation group was compared to a control group without supplementation. Results showed no significant differences in lower back pain (VAS), spine function (ODI and RMDQ), or quality of life (36-item short-form health survey) between groups at baseline or four-to-six weeks post-supplementation. However, at 10–12 weeks and 22–26 weeks post-supplementation, the supplementation group showed greater improvement in VAS scores, functional outcomes, and quality of life [6]. Notably, both the supplementation and non-supplementation groups received additional pain treatments during the study, including pregabalin 50 mg twice daily and Limaprost (an oral prostaglandin E1 analog), one tablet three times daily.

The remaining three studies on vitamin D supplementation found it to be no more effective than a placebo for LBP [7–9]. Importantly, a 2018 systematic review and meta-analysis concluded that prescribing vitamin D for LBP requires additional well-designed and sufficiently powered studies before it can be officially recommended by providers [8].

#### B vitamin supplementation

Three studies investigated the use of B vitamins, with or without other supplements or treatment measures. The study designs included a systematic review and meta-analysis (*n* ═ 1), a randomized, double-blind, parallel-group prospective study (*n* ═ 1), and a non-randomized, prospective study (*n* ═ 1).

The systematic review and meta-analysis examined the effect of combining diclofenac with B vitamins (thiamine, pyridoxine, and cyanocobalamin) on LBP [10]. Five studies were included, each comparing the efficacy of diclofenac combined with B vitamins against diclofenac monotherapy (control). The primary outcome measure was the patient’s VAS score. The study concluded that diclofenac supplementation with thiamine, pyridoxine, and cyanocobalamin may improve outcomes for patients with acute LBP compared to diclofenac monotherapy. However, the authors reported there is not enough evidence to recommend this treatment regimen for all types and durations of LBP [10].

A randomized, double-blind, parallel-group prospective study investigated the effects of 1.5 mg uridine triphosphate trisodium + 2.5 mg cytidine monophosphate disodium + 1000 µg hydroxocobalamin (Group A) vs 100 mg thiamine + 100 mg pyridoxine + 5000 µg cyanocobalamin (Group B) for the treatment of LBP [11]. There were 317 patients in each treatment group. The authors found a superior VAS reduction at 30 days of treatment in Group A compared to Group B (--4.5; 95% CI: --7.2, --1.8; *P* < 0.0001). However, VAS findings were equivalent between both treatment groups at 60 days of treatment. Furthermore, the RMDQ results improved in both groups at day 30 (2.3 points [± 3.0; 95% CI: 1.9, 2.6] for Group A vs 3.3 points [± 3.8; 95% CI: 2.9, 2.7] for Group B) and 60 (Group A mean score 0.9 ± 1.8 [95% CI: 0.7, 1.1] vs Group B mean score 1.3 ± 2.5 [95% CI: 1.1, 1.6]) [11]. Compared to baseline pretreatment scores in both treatment groups, score improvements within each group were statistically significant (*P* < 0.0001). Notably, 374 adverse events were reported during the treatment period, with 35.6% (*n* ═ 133) of the total events occurring in treatment Group A and 65.4% (*n* ═ 241) in treatment Group B. Adverse events included but were not limited to, headache, hypokalemia, nausea, hot flushes, muscle cramps, muscle weakness, loss of appetite, and vomiting [11].

A third non-randomized prospective study investigated operative vs nonoperative (including nutritional supplementation) treatments for 134 patients suffering from lumbar spine degenerative diseases with canal and/or foraminal stenosis [12]. Patients received either spine surgery (i.e., laminectomy, partial laminectomy, or hemilaminectomy) or multidisciplinary treatment consisting of physiotherapy, dietitian support, and supplementation with thiamine, pyridoxine, cyanocobalamin, and Neuracalm (pregabalin 75 mg + methylcobalamin 750 mcg) twice daily. The operative arm included 65 patients, and the nonoperative arm included 69 patients. Outcomes were measured using the Swiss Spinal Stenosis Questionnaire. Outcomes (mean ± SD) were slightly better in the nonoperative group (48.1 ± 8.2) compared to the operative group (47.9 ± 9); however, the results were not statistically significant [12].

#### Other LBP studies

The remaining LBP studies (*n* ═ 13) covered a wide range of nutritional therapies and supplements [13–25]. Outside of the two alpha lipoic acid (ALA) studies elaborated upon below, no specific themes or trends were identified within the remaining LBP studies.

Two studies involved ALA. The first ALA study was a prospective, randomized, open-label study in which group I received pulsed radiofrequency of the dorsal root ganglion for chronic lumbosacral radicular pain, while group II received the same treatment in addition to oral 600 mg ALA three times daily for three weeks, followed by 600 mg daily for two weeks [26]. Patients were evaluated using the NRPS and ODI. Results at three months, according to NRPS, were as follows: Group I pre-procedure NRPS ═ 8.0 (6–9); post-procedure ═ 4.0 (1–6); Group II pre-procedure ═ 8.0 (7–9); post-procedure ═ 3.0 (0–6) with *P* value ═ 0.005 [26]. At six-months post-procedure, NRPS results were: Group I pre-procedure NRPS ═ 8.0 (6–9); post-procedure ═ 4.0 (2–6); Group II pre-procedure ═ 8.0 (7–9); post-procedure ═ 3.0 (2–6) with *P* value ═ 0.011 [26]. The authors concluded that ALA, in addition to pulsed radiofrequency of the dorsal root ganglion, can be useful for treating lumbosacral radicular pain.

The second ALA study was a prospective, non-randomized, open-label study of 98 adult patients with chronic LBP with or without radiculopathy [27]. Patients were treated with a combination of 600 mg ALA and 140 UI superoxide dismutase (SOD) daily. Patients were evaluated using the RMDQ and pain rating scale (PRS). At study completion, only 8.2% of patients continued to utilize analgesics vs 73.5% who utilized analgesics at baseline (*P* < 0.01) [27]. Additionally, there was a statistically significant improvement in both PRS and RMDQ scores 40 days post-therapy (*P* < 0.05) [27]. Therefore, the authors conclude that the combination of oral treatment with ALA and SOD improves functionality and reduces the use of analgesics in chronic LBP patients.

### Neck pain

Only a single clinical study focused specifically on neck pain. This study, involving 260 northern Chinese sedentary office workers, investigated the effects of black soybean on chronic cervical pain [28]. Participants were divided into groups that consumed either 1, 5, or 10 g of cooked black soybean at breakfast, lunch, and dinner, amounting to a total daily intake of 3, 15, or 30 g of black soybean, respectively. The investigators found that VAS, NDI, and pain scores significantly decreased in participants who consumed 15 or 30 g of black soybean daily compared to the 3 g black soybean daily group (*P* < 0.05). Furthermore, the 30 g black soybean daily group showed better relief from chronic cervical pain as compared to the 15 g black soybean group (*P* < 0.05) [28].

### Generalized back pain

Two studies did not specifically report the targeted spinal area (i.e., cervical, thoracic, or lumbar). Therefore, the term “generalized” back pain is used for these studies. The first study compared the effects of calcium carbonate and ossein-hydroxyapatite complex on generalized back and knee pain, as well as quality of life, in osteopenic perimenopausal women [29]. This was a randomized, open-label, parallel-group, controlled, prospective study. A total of 74 perimenopausal women were randomized to either a group taking 1200 mg/day of calcium carbonate (control) or a group taking 1660 mg/day of ossein-hydroxyapatite complex (study). Back and knee pain were recorded using the VAS, and the visual rating system (VRS) was used to measure exercise-induced back and knee pain. Changes in quality of life were also assessed using the Medical Outcome Study Short Form-36 (SF-36). The investigators found a significant analgesic effect of the ossein-hydroxyapatite complex, with a notable reduction in mean VAS and VRS pain scores in the study group after five (2.18 [0.58] *P* < 0.001) and six (1.97 [0.59] *P* < 0.001) months of treatment. Furthermore, as compared to the calcium carbonate group, the ossein-hydroxyapatite complex group showed improvements in physical (0.36 [0.30] vs 0.44 [0.31] *P* value 0.294) and mental (-0.02 [0.68] vs 0.17 [0.54] *P* value 0.213) quality of life, as measured by the SF-36, at six months [29].

The second study, a parallel-group, randomized, double-blind, placebo-controlled trial, investigated vitamin D supplementation for generalized back pain disability in vitamin D-deficient, overweight, or obese adults [30]. This study included 65 overweight or obese adults, with vitamin D deficiency, who were randomized to receive either a bolus oral dose of 100,000 IU followed by 400 IU of cholecalciferol per day or a matching placebo for 16 weeks. The authors found that vitamin D supplementation had no significant impact on back pain intensity or disability in overweight or obese patients with vitamin D deficiency. Self-reported measures included the Chronic Pain Grade questionnaire for back pain assessment and the International Physical Activity Questionnaire. However, among overweight or obese patients with severe vitamin D deficiency at baseline (25[OH]D < 30 nmol/L), there was a significant reduction in back pain disability scores in the vitamin D group compared to placebo (*b* [95% CI] ═ –11.6 [-22.4, -0.8], *P* ═ 0.04) [30].

## Discussion

In this review, vitamins D and B supplementation for LBP were the most strongly supported interventions. However, three of the eight vitamin D studies found it to be no more effective than a placebo for LBP. The studies favoring vitamin D supplementation for LBP varied in study duration and mode of supplementation. One study instructed patients to take an active vitamin-D3 sachet of 60,000 IU orally mixed in a glass of milk, while another compared 60,000 IU vitamin D in granules, nano syrup, or soft gel capsules [2, 6]. The mode of supplementation and the instructions given to patients may have resulted in significant differences in vitamin D absorption, which could have impacted study outcomes.

In the vitamin B studies, variations in study design (i.e., systematic review and meta-analysis [*n* ═ 1]; randomized, double-blind, parallel-group prospective study [*n* ═ 1]; and non-randomized prospective study [*n* ═ 1]) make it impossible to draw definitive conclusions regarding the efficacy of vitamin B supplementation for LBP. Furthermore, each of the included vitamin B studies investigated different B vitamins (i.e., methylcobalamin, thiamine, pyridoxine, hydroxycobalamin, and cyanocobalamin) and doses. For example, one study investigated a Neuracalm supplement containing pregabalin 75 mg and methylcobalamin 750 mcg. It is possible that pregabalin 75 mg alone accounted for the LBP relief, regardless of the addition of methylcobalamin 750 mcg.

To further investigate the usefulness of nutritional supplementation for spine-related pain, future research studies should be designed to isolate the target nutrient of concern. For example, the nutritional supplement should not be consumed alongside or combined with other supplements or foods that could confound study outcomes and results. Additionally, future studies should utilize consistent outcome measures for spine-related pain and functional improvement to allow for meaningful comparisons between studies. The studies included in this review varied in their use of instruments to measure both patient pain and functionality. Lastly, it is worth noting that several of the included studies did not collect baseline patient bloodwork, making it impossible to determine how the nutritional strategies or targeted supplementation impacted potential deficiencies. Future studies should include baseline laboratory values to show the impact of targeted nutritional supplementation more clearly.

## Limitations

This study’s findings are limited by its stringent inclusion criteria. Future reviews could consider including additional study designs, languages, supplements, or nutritional strategies. Additionally, future research should consider including studies published outside the past ten years and screening references to identify additional studies that may have been missed during the primary search.

## Strengths

While the stringent inclusion criteria are a limitation, they also represent a strength of this study. To the authors’ knowledge, this is the first scoping review on nutritional supplementation or strategies for spine-related pain. As this topic grows in popularity among professionals in orthopedics, physical medicine and rehabilitation, interventional pain, nutrition and dietetics, and other subspecialties, the evidence base is relevant to a wide audience of practitioners.

## Conclusions

There is a wide range of published research on nutritional supplements and strategies related to spine pain. However, many studies were conducted with suboptimal designs, small patient sample sizes, and have not been replicated in follow-up research. In this scoping review, the largest bodies of evidence were found for vitamin D and B vitamin supplementation in LBP conditions. Conflicting findings were reported for both vitamins, indicating that further research is necessary before they can be confidently recommended to patients suffering from LBP.

## Supplemental data

Table S1 is available at the following link:

https://www.bjbms.org/ojs/index.php/bjbms/article/view/11393/3582

Table S2 is available at the following link:

https://www.bjbms.org/ojs/index.php/bjbms/article/view/11393/3581
